# New insights on the potential effect of vinpocetine in Parkinson’s disease: one of the neglected warden and baffling topics

**DOI:** 10.1007/s11011-023-01254-y

**Published:** 2023-06-19

**Authors:** Hayder M. Al-kuraishy, Athanasios Alexiou, Marios Papadakis, Omnya Elhussieny, Hebatallah M. Saad, Gaber El-Saber Batiha

**Affiliations:** 1 Department of Pharmacology, Toxicology and Medicine, Medical Faculty, College of Medicine, Al- Mustansiriyah University, P.O. Box 14132, Baghdad, Iraq; 2 Department of Science and Engineering, Novel Global Community Educational Foundation, Hebersham, NSW 2770 Australia; 3 AFNP Med, 1030 Wien, Austria; 4 Department of Surgery II, University Hospital Witten-Herdecke, University of Witten-Herdecke, Heusnerstrasse 40, Wuppertal, Germany; 5 Department of Histology and Cytology, Faculty of Veterinary Medicine, Matrouh University, 51744 Marsa Matruh, Egypt; 6 Department of Pathology, Faculty of Veterinary Medicine, Matrouh University, Marsa Matruh, 51744 Egypt; 7 Department of Pharmacology and Therapeutics, Faculty of Veterinary Medicine, Damanhour University, Damanhour, 22511 Egypt

**Keywords:** Vinpocetine, Parkinson's disease, Neuroinflammation

## Abstract

Vinpocetine (VPN) is an ethyl apovincaminate that has anti-inflammatory and antioxidant effects by inhibiting the expression of nuclear factor kappa B (NF-κB) and phosphodiesterase enzyme 1 (PDE-1). VPN is used in the management of stroke, dementia, and other neurodegenerative brain diseases. VPN may be effective in treating Parkinson’s disease (PD). Therefore, this review aimed to clarify the mechanistic role of VPN in the management of PD. VPN has protective and restorative effects against neuronal injury by reducing neuroinflammation, and improvement of synaptic plasticity and cerebral blood flow. VPN protects dopaminergic neurons by reducing oxidative stress, lipid peroxidation, glutamate neurotoxicity, and regulation of Ca^+ 2^ overloads. VPN can alleviate PD neuropathology through its anti-inflammatory, antioxidant, antiapoptotic and neurogenic effects. VPN through inhibition of PDE1 improves cyclic adenosine monophosphate (cAMP)/cyclic guanosine monophosphate (cGMP) signaling in the dopaminergic neurons of the substantia nigra (SN). VPN improves PD neuropathology through PDE1 inhibition with a subsequent increase of the cAMP/cGMP signaling pathway. Therefore, increasing cAMP leads to antioxidant effects, while augmentation of cGMP by VPN leads to anti-inflammatory effects which reduced neurotoxicity and development of motor severity in PD. In conclusion, this review indicated that VPN could be effective in the management of PD.

## Introduction

**Fig. 1:**
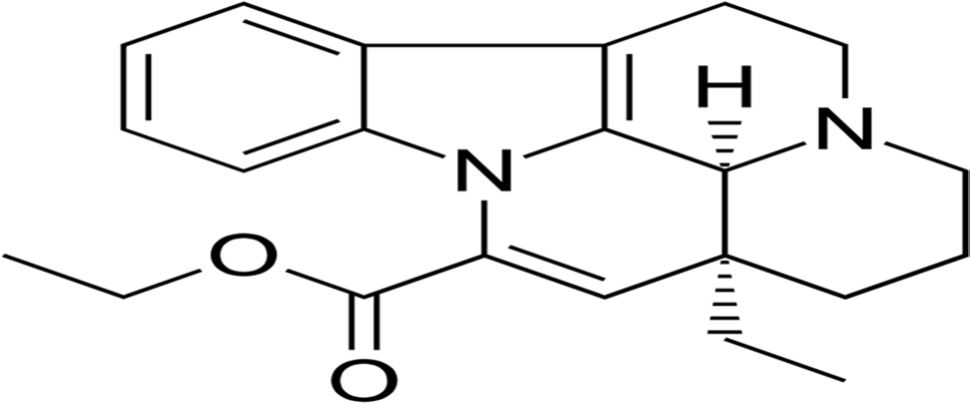
Chemical structure of Vinpocetine

Vinpocetine (VPN) is an ethyl apovincaminate (Fig. 1) derived from vinca alkaloid vincamine extracted from Vocanga Africana seeds and Vinca minor leaves (Al-Kuraishy et al. 2020).

VPN was first used in 1978 to treat dementia, stroke, and memory disorders (Patyar et al. 2011). It has been extensively used to manage dementia and stroke in European and Asian countries. In addition to its usage as a nootropic and dietary supplement; however, it was not approved for therapeutic use in the USA (Al-Kuraishy et al. 2019).

The mechanism of VPN is related to the inhibition of sodium channels, reduction of calcium influx, and antioxidant effects (Al-kuraishy and Al-Gareeb 2020). VPN is regarded as a potent inhibitor of phosphodiesterase enzyme 1 (PDE-1) (Patyar et al. 2011). Besides, VPN has an anti-inflammatory effect by inhibiting the expression of nuclear factor kappa B (NF-κB) via stabilization of IκB, an inhibitor of NF-κB. VPN increases dihydroxyphenylacetic acid (DOPAC), a metabolic product of dopamine that suggests VPN’s role in the metabolism of dopamine (Zhang et al. 2018). Moreover, VPN has anti-platelet activity, thereby improving brain blood flow and brain metabolism (Medina 2010).

VPN is considered a safe drug, though it is contraindicated in pregnancy (Medina 2010). Prolonged use of VPN is associated with the development of some side effects, including hypotension, tachycardia, dizziness, dry mouth, nausea, heartburn, and flushing (Al-Kuraishy et al. 2020). VPN has a specific pharmacokinetic profile; the effective therapeutic dosage of VPN is 5–10 mg (Medina 2010). VPN half-life is 1–2 h; it is highly absorbed from the intestine with 56.6% bioavailability, peak plasma level is reached after one hour of oral administration, highly distributed, crosses the blood-brain barrier (BBB), metabolized by the liver and excreted by urine (Bönöczk et al. 2000; Medina 2010; Patyar et al. 2011).

VPN is effective in managing neurodegenerative brain diseases, including Parkinson’s disease (PD) (Ping et al. 2019). Thus, this review aims to clarify the mechanistic role of VPN in PD.

## Parkinson’s disease

**Fig. 2:**
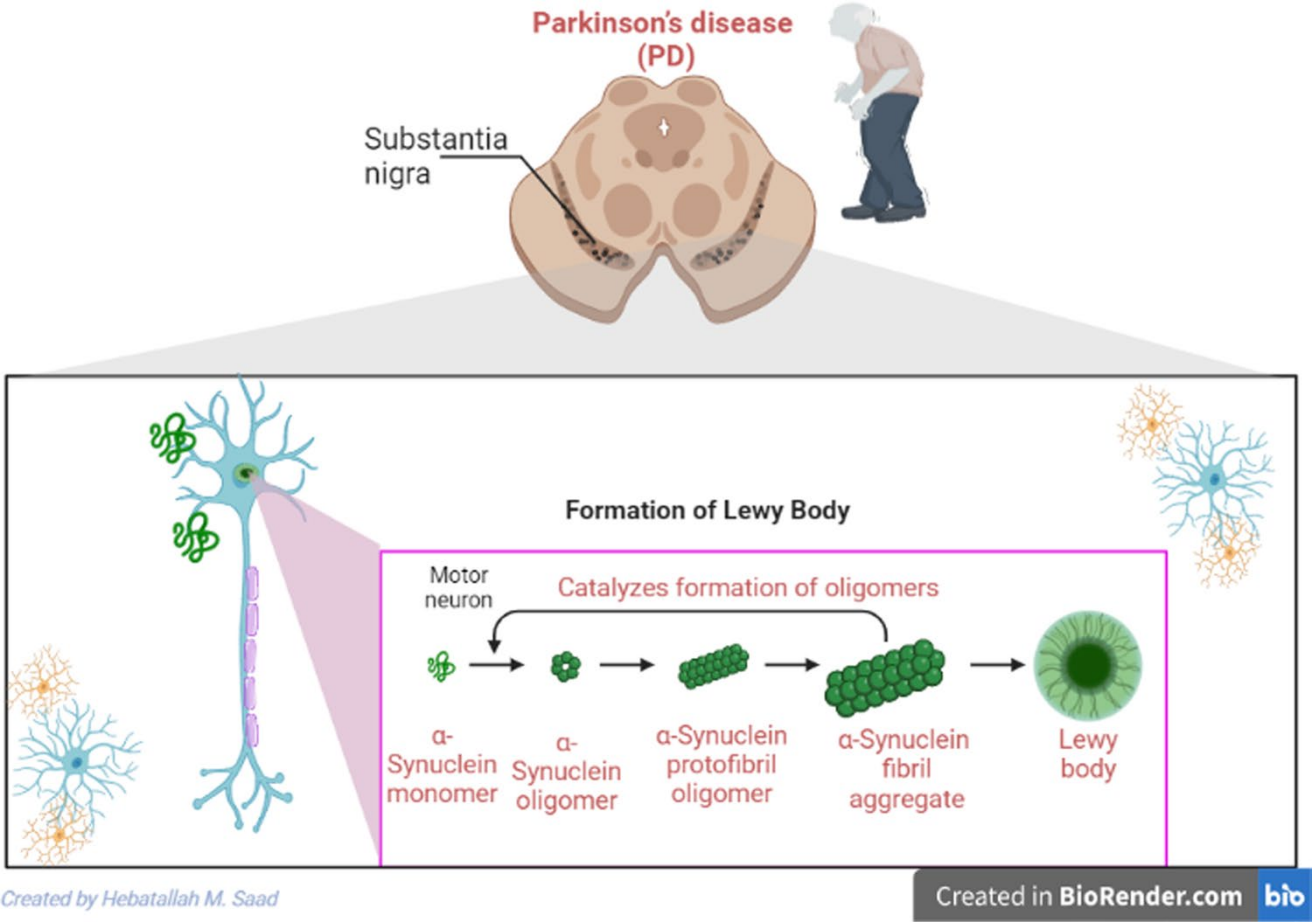
Pathophysiology of Parkinson’s disease (PD)

PD is the second most common neurodegenerative brain disease, following Alzheimer’s disease (AD) (Alrouji et al. 2023; Poewe et al. 2017). PD was first recognized in 1817 by Doctor James Parkinson, who described shaking palsy (Savica et al. 2010). PD is developed due to dopaminergic neuron loss in the substantia nigra (SN) with subsequent significant dopamine deficiency in the caudate nucleus and putamen (Savica et al. 2010). These pathological changes lead to motor dysfunctions, including rigidity, resting tremors, bradykinesia and walking difficulty (Savica et al. 2010). In PD, many non-motor disorders include apathy, depression, anxiety, autonomic disorders, dementia, neuropsychiatric disorders, cognitive dysfunction, and sleep disturbances (Thenganatt and Jankovic 2014). The incidence of PD in the general population is 0.3% and reaches 4% above the age of 80 years (Savica et al. 2016). The mean age of PD onset is around 60 years; however, new-onset PD may develop in the younger age group of 20–50 years (Patyar et al. 2011). The annual incidence of PD is 8–18 per 100,000 (Savica et al. 2016). Males are more affected than females by PD, with a ratio of 3:2 (Savica et al. 2016). In 2040, the number of PD patients will extend to 14 million people at risk for the development of the Parkinson’s pandemic (Savica et al. 2016). PD may be genetic due to mutations of 9 autosomal recessive and 11 autosomal dominant, and non-genetic due to exposure to pesticides, carbon disulfide and manganese (Sardi et al. 2018). The neuropathological characteristic of PD is the deposition of Lewy bodies from aggregated α-synuclein (Fig. 2) (Stefanis 2012).

The deposition of α-synuclein is not limited to the SN but throughout the entire brain, such as the autonomic nervous system (ANS) (Stefanis 2012). Deposition of α-synuclein is progressive for many years before the development of a symptomatic period (Stefanis 2012). Deposition of α-synuclein is started initially in the ANS, mostly in the dorsal motor nucleus of glossopharyngeal and vagus nerves and then spreads to the other brain areas in stage I of PD neuropathology (Sharabi et al. 2021). Stage II α-synucleinopathy is propagated by spreading to brain stem areas, including medulla oblongata, locus coeruleus and pontine tegmentum. In stage III α-synucleinopathy, the SN is affected. During stage IV α-synucleinopathy, there is profound degeneration of dopaminergic neurons in the SN and pathology of Lewy bodies extended to the temporal cortex. In the last V and VI stages, Lewy bodies are highly deposited in the neocortex leading to the development of cognitive dysfunction (Niwa et al. 2011). These verdicts proposed that PD neuropathology is progressive and not limited to SN degeneration. Notably, in the prodromal phase, non-motor symptoms, including anosmia, constipation, sleep disorders, and depression, develop before dopaminergic degeneration in the SN. Following the development of motor symptoms due to dopaminergic degeneration in the SN, cognitive dysfunctions are propagated due to the involvement of the temporal cortex (Wakabayashi 2020).

Novel treatment for the management of PD is required. The management of PD should be broader than symptomatic improvement, as treatment of non-motor symptoms remains challenging and not traditional. However, early detection of PD neuropathology before the development of excitotoxicity and apoptosis may prevent symptomatic PD. Though, most PD in animal model studies reflects the acute status of dopaminergic neuron degeneration regardless of PD neuropathology stages (Biswas and Bagchi 2023). Recently, it has been shown that nicotine, caffeine and some natural products like polyphenols in green tea prevent mutation in the parkin protein, which is involved in the pathogenesis of PD (Biswas and Bagchi 2023). Interfering with aggregation of α-synuclein like nicotine and caffeine could prevent PD (Biswas and Bagchi 2023). Furthermore, inosine, a urate precursor, may prevent dopaminergic neuron degeneration in the SN (Bluett et al. 2021). In addition, many preclinical studies have proposed immunotherapy against α-synuclein by either active or passive immunizations (Poewe et al. 2021). Of interest, modulators of α-synuclein aggregations have been suggested to be effective in the prevention of PD (Al-kuraishy et al. 2023; Wang et al. 2021). VPN could be of interest in managing PD because of its anti-inflammatory and antioxidant activities.

## Neuroprotective effects of VPN

VPN has protective and restorative effects against neuronal injury by reducing neuroinflammation and synaptic plasticity and improving cerebral blood flow in acute ischemic stroke (Bönöczk et al. 2000). VPN has been reported to be effective against stroke, chronic cerebral insufficiency, and cerebral atherosclerosis (Cai et al. 2013). VPN attenuates endothelial dysfunction and lipid accumulation by inhibiting macrophage activity and oxidation of low-density lipoprotein (LDL) (Cai et al. 2013). VPN also effectively manages epilepsy and dementia through the modulation of synaptic plasticity and neurotransmission (Meador et al. 2021). A recent randomized clinical trial by Garza-Morales et al. (2019) showed that VPN effectively treated focal epilepsy. An experimental study confirmed that VPN effectively alleviated cerebral ischemic reperfusion (I/R) injury and associated neuroinflammation by inhibiting microglia activation (Wang et al. 2014). A pilot single-blind clinical trial illustrated that VPN improved clinical outcomes in patients with acute ischemic stroke (Feigin et al. 2001).

Moreover, VPN improves regional brain blood flow via redistribution of cerebral blood flow (CBF) in patients with chronic cerebral ischemia (Szilágyi et al. 2005). A double-blind imaging study involving 13 patients with chronic ischemia revealed that VPN increased regional and global cerebral blood flow (Szilágyi et al. 2005). Thus, VPN had a neuroprotective effect via redistribution of cerebral blood flow. VPN regulates blood viscosity and increases CBF modulation of erythrocyte deformability and vasodilation in patients with acute ischemic stroke (Panda et al. 2022). A systematic review and meta-analysis involving four clinical trials of 236 patients on VPN and placebo showed that VPN was effective in patients with acute ischemic stroke without reduction of case fatality (Panda et al. 2022). VPN regulates the neuroplastic process by controlling glial activity and glucose uptake (Xu et al. 2021). Moreover, VPN improves cognitive function in patients with AD and epilepsy (Valikovics et al. 2012).

**Fig. 3:**
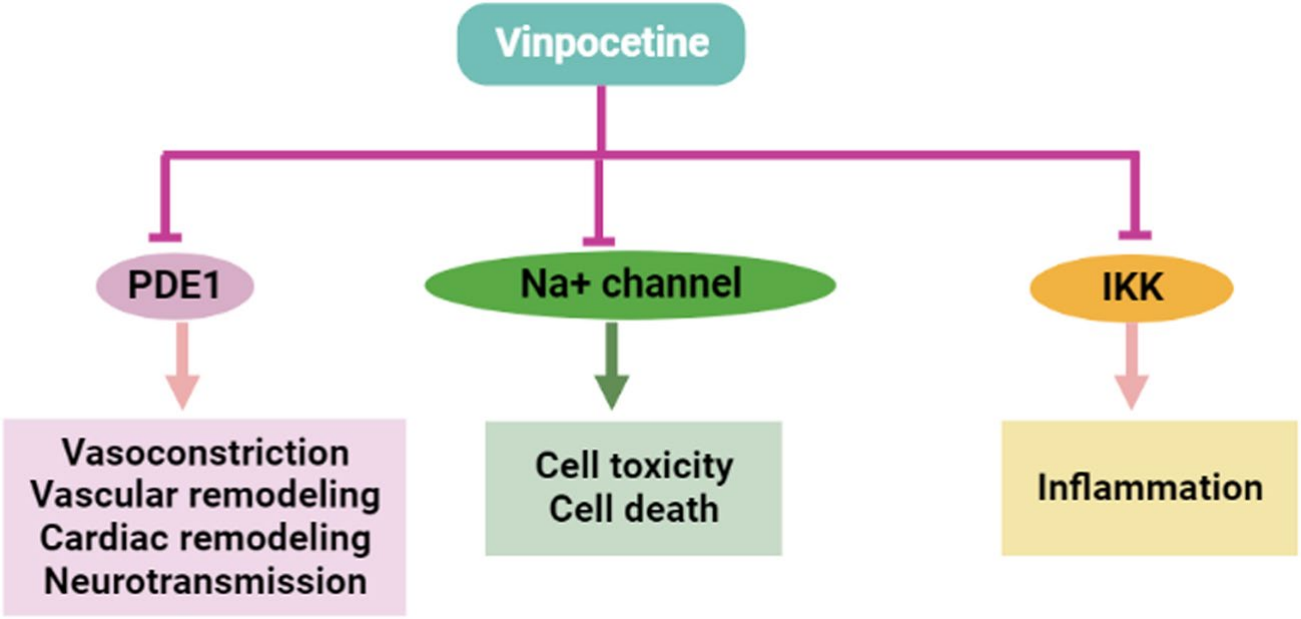
Neuroprotective activity of VPN

Furthermore, VPN reduces the expression of NF-κB and the release of pro-inflammatory cytokines after I/R, thereby preventing the development of brain edema (Wang et al. 2014). In vitro study observed that VPN had a protective activity on cortical neurons by preserving mitochondrial function via regulation of mitochondrial membrane potential (MMP) (Tárnok et al. 2008). VPN has been reported to interfere with various signaling cascades during brain ischemic events, including activation of voltage-gated Na^+^ channel, adenosine triphosphate (ATP) depletion, glutamate excitotoxicity and Ca^+ 2^ dysregulation (Hadjiev 2003). VPN has potent antioxidant and anti-inflammatory effects that attenuate BBB dysfunction during brain ischemic events (Hadjiev 2003). These findings suggest VPN’s universal role in managing different neurological disorders (Fig. 3).

## Role of VPN in PD

Numerous studies confirmed the protective effects of VPN against the development of PD. For example, an experimental study by Zaitone et al. (2012) showed that VPN attenuated rotenone-induced PD in rats. VPN protects dopaminergic neurons by reducing oxidative stress, lipid peroxidation, glutamate neurotoxicity and regulation of Ca^+ 2^ overload (Zaitone et al. 2012). Likewise, pre-treatment with VPN reduced the rotenone-induced locomotor deficit, motor incoordination, and cognition deficits in rats (Ishola et al. 2023). In addition, rotenone-induced a significant increase in the level of interleukin (IL)-6, tumor necrosis factor-alpha (TNF-α), oxidative stress markers, cholinergic signaling, guts dysfunction and hematologic dysfunctions which were attenuated by VPN administration. Immunostainings showed that rotenone-induced dopamine neuron loss, microglia reactivity, astrocytes activation, toll-like receptor4 (TLR4) and α-synuclein expressions were attenuated by VPN administration (Ishola et al. 2023). VPN also attenuates paraquat-induced PD in mice by improving antioxidant defense mechanisms and inhibiting neuroinflammation (Ishola et al. 2018). These preclinical findings indicated that VPN can alleviate PD neuropathology through its anti-inflammatory, antioxidant, antiapoptotic and neurogenic effects (Abu-Elfotuh et al. 2022).

A recent clinical study involving 89 PD patients compared to 42 healthy controls observed that VPN was influential in managing cognitive impairment in PD patients (Ping et al. 2019). The cognitive enhancing effect of VPN is related to its potent anti-inflammatory effects. Circulating levels of TLR and pro-inflammatory cytokines were reduced in PD patients following treatment with VPN (Ping et al. 2019). The anti-inflammatory role of VPN in the treatment of PD. VPN regulates TLR2/3/4 mRNA and protein expression; moreover, it modulates downstream signaling proteins and cytokines. However, there is no stable or clear cause-effect relationship between its anti-inflammatory action and cognitive enhancement. Thus, VPN is a good candidate for future therapy in PD patients (Ping et al. 2019).

These observations indicated that VPN has a neuroprotective effect against the development and progression of PD neuropathology.

## Mechanistic role of VPN in PD

### Neuroinflammation

Neuroinflammation transcription factors and are involved in the pathogenesis and progression of PD (Tiwari and Pal 2022). Neuroinflammation has been recognized as a cornerstone in the progression of neurodegenerative diseases and especially in the chronic loss of nigral dopaminergic neurons in PD. Noteworthy, the generation of reactive oxygen species (ROS) induces inflammation through the activation of NF-кB activation which in turn led to enhanced production of downstream proinflammatory mediators (Hirsch et al. 2012). Lipopolysaccharide (LPS) activation adjacent to the dopaminergic neurons in the SN increases neuronal loss and degeneration (Tiwari and Pal 2022). Postmortem analysis of brain tissues and cerebrospinal fluid of PD patients demonstrated that NF-κB, mechanistic target of rapamycin (mTOR), Ying-Yang1 (YY1) and fibroblast growth factor 20 (FGF20) were over-expressed and associated with the dopaminergic neuronal loss (Tiwari and Pal 2022). Both pro-inflammatory cytokines and chemokines are linked with dopaminergic degeneration in the SN with the propagation of PD (Hirsch et al. 2012; Tiwari and Pal 2022). VNP had a protective effect against pilocarpine-induced seizures by inhibiting the mTOR pathway in rats (El-Sayed et al. 2021). Therefore, VNP is a valuable candidate for epilepsy therapy via its modulation of the mechanisms underlying epileptogenesis with emphasis on its modulatory effect on the mTOR signaling pathway (El-Sayed et al. 2021). VPN has been observed to inhibit neuroinflammation in preclinical and clinical studies (Zhou et al. 2020). VPN in hypoxic mice inhibits inflammatory molecules including IL-6 and TNF-α which are involved in the development of neuroinflammation, indicating that VPN as a unique anti-inflammatory agent may be beneficial for the treatment of neuroinflammatory diseases (Zhao et al. 2011). In vitro study demonstrated that VPN inhibits microglia activation induced by LPS (Zhou et al. 2020). In vitro study demonstrated that VPN inhibits the production of nitirc oxide (NO) and inflammatory factors such as IL-1β, IL-6 and TNF-α in BV-2 microglia, in which cells were treated with LPS by suppressing the expression of translocator protein (TSPO). As well, VPN blocks the expression of Akt, Junk, p38, NF-κB and activator protein-1 (AP-1) in LPS-stimulated microglia, indicating that VPN has an anti-inflammatory effect by partly targeting NF-κB/AP-1 (Zhao et al. 2011). VPN attenuates the release of pro-inflammatory cytokines and the development of neuroinflammation via the activation of adenosine monophosphate protein kinase (AMPK) (Zhou et al. 2020). AMPK is a crucial regulator of immunity and inflammation by inhibiting TLR4 and NF-κB (Zhou et al. 2020). Moreover, the nucleotide-binding oligomerization domain-, leucine-rich repeat and pyrin domain-containing 3 (NLRP3) inflammasome is an inflammatory complex existing in microglia (Batiha et al. 2022). Its activation promotes the secretion of the inflammatory cytokine interleukin-1β/18 (IL-1β/18) and induces pyroptosis, a type of cell death that possesses the potential for inflammation, to rupture microglia to further release IL-1β (Wang et al. 2019). NLRP3 inflammasomes play a crucial role in PD via caspase 1 activation, primarily induced by mitochondrial damage. NLRP3 binds to apoptosis-associated speck-like protein and forms inflammasomes in the brain. Inflammasomes act as a platform for caspase 1 to induce IL1β maturation, leading to neuronal pyroptosis (Sarkar et al. 2017). Moreover, α-synuclein also activates NLRP3 inflammasomes. Mutations to PRKN (encoding Parkin) are the most common cause of autosomal recessive familial and sporadic early-onset PD. Evidence has confirmed a relationship between Parkin and NLRP3 inflammasomes (Fan et al. 2020). It has been shown that VPN has ability to inhibit the expression of NLRP3 inflammasomes in mice with age-related macular degeneration (Liu et al. 2014). As well, VPN alleviates ischemic stroke by regulating levels of NLRP3 inflammasome, and proinflammatory cytokines in mice, offering an alternative medication for ischemic stroke associated with inflammation (Han et al. 2021).

These findings pointed up that VPN attenuates neuroinflammation-induced PD by inhibiting the expression of inflammatory signaling NF-κB/ NLRP3 inflammasome and the release of pro-inflammatory cytokines. These verdicts confirmed that VPN can attenuate neuroinflammation which is involved in the pathogenesis of PD.

### Oxidative stress

**Fig. 4:**
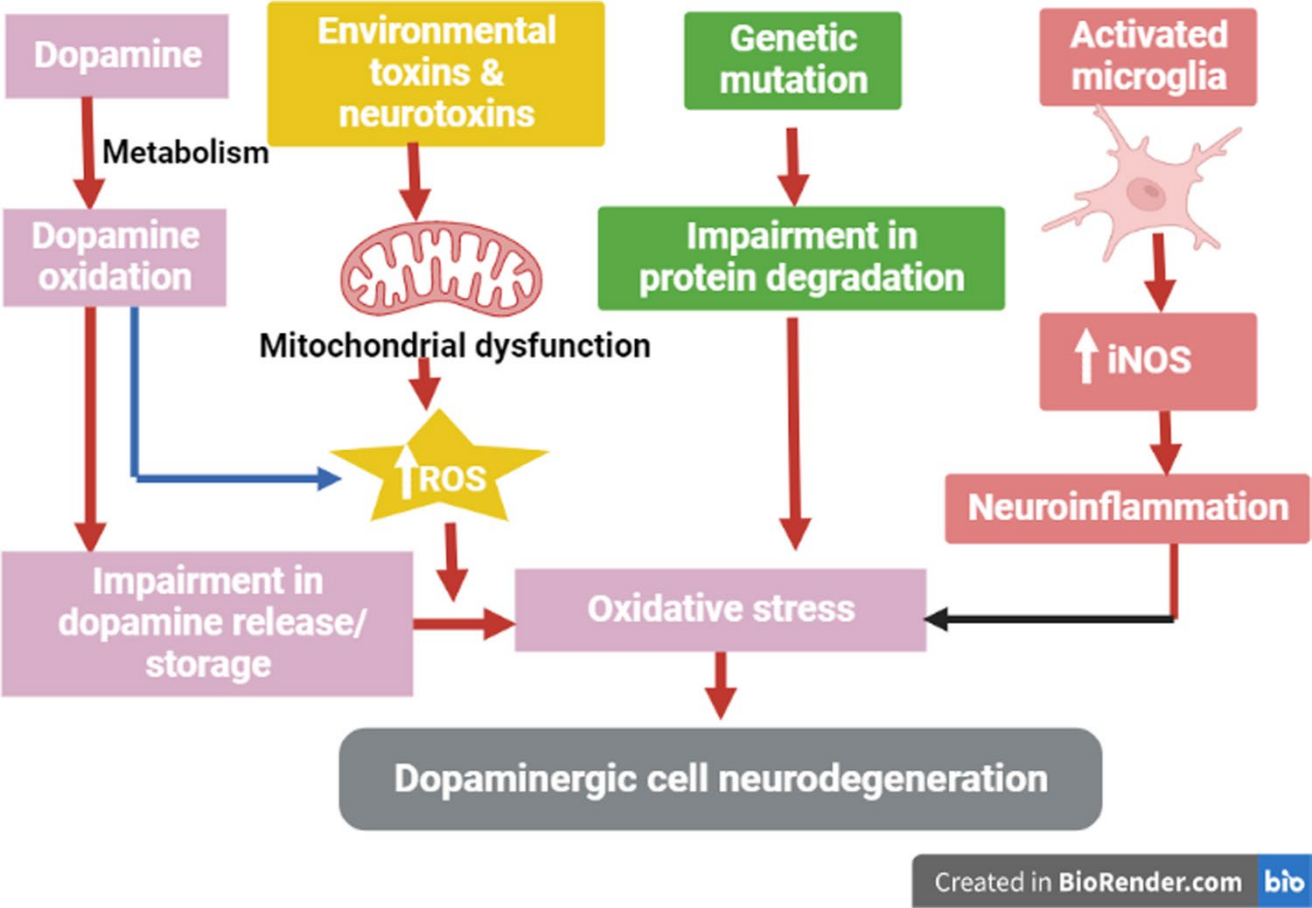
Oxidative stress and dopaminergic neurons degeneration. ROS: reactive oxygen species; iNOS: inducible nitric oxide synthase

Mitochondrial dysfunction, the release of ROS and the development of oxidative stress are involved in the pathogenesis of PD (Henchcliffe and Beal 2008). Oxidative stress is believed to play a critical role in dopaminergic neurotoxicity (Blesa et al. 2015). In PD, mitochondrial dysfunction is the primary source of ROS linked with dopaminergic neurotoxicity (Blesa et al. 2015). Dysfunction of different pathways due to genetic variations increases the risk of oxidative stress due to mitochondrial dysfunction (Blesa et al. 2015). Oxidative stress promotes aggregation of α-synuclein with a reduction of intracellular ATP production and development of dopaminergic neuron degeneration (Fig. 4). Of note, α-synuclein overexpression promotes the formation of α-synuclein-immunopositive inclusion-like structures and mitochondrial alterations accompanied by increased levels of free radicals (Han et al. 2021). These alterations are ameliorated by pretreatment with antioxidants such as vitamin E (Hsu et al. 2000). Taken together these results suggest that abnormal accumulation of α-synuclein could lead to mitochondrial alterations that may result in oxidative stress and, eventually, cell death. A systematic review and meta-analysis revealed that PD is accompanied by increased oxidative stress (Wei et al. 2018). Different studies revealed that VPN has antioxidant effects (Deshmukh et al. 2009; Santos et al. 2000). VPN can act as an antioxidant and prevent the formation of ROS and lipid peroxidation in rat brain synaptosomes (Santos et al. 2000). The antioxidant effect of VPN might contribute to the protective role exerted by reducing neuronal damage in pathological situations (Santos et al. 2000). Chronic treatment with VPN also reduced significantly the increase in acetylcholinesterase activity and lactate dehydrogenase levels indicating the restorative capacity of VPN concerning cholinergic functions and preventing the neuronal damage (Deshmukh et al. 2009). The observed beneficial effects of VPN on spatial memory may be due to its ability to favorably modulate cholinergic functions, prevent neuronal cell damage and possibly through its antioxidant mechanism (Deshmukh et al. 2009). VPN inhibits oxidative stress by scavenging free radicals, prevention of Glutathione (GSH) depletion, reducing superoxide anion and nitric oxide production, and lipid peroxidation (Ruiz-Miyazawa et al. 2015). Moreover, VPN prevents paraquat-induced motor deficits, memory impairment, oxidative stress, and neuroinflammation through the enhancement of the antioxidant defense system and the inhibition of neuroinflammatory cytokine (Ishola et al. 2018). Furthermore, VPN attenuates demyelination in the brain rats by inhibiting oxidative stress and lipid peroxidation (Ruiz-Miyazawa et al. 2015). These findings pointed out that VPN by its antioxidant effect can mitigate PD neuropathology.

### PDEs

**Fig. 5:**
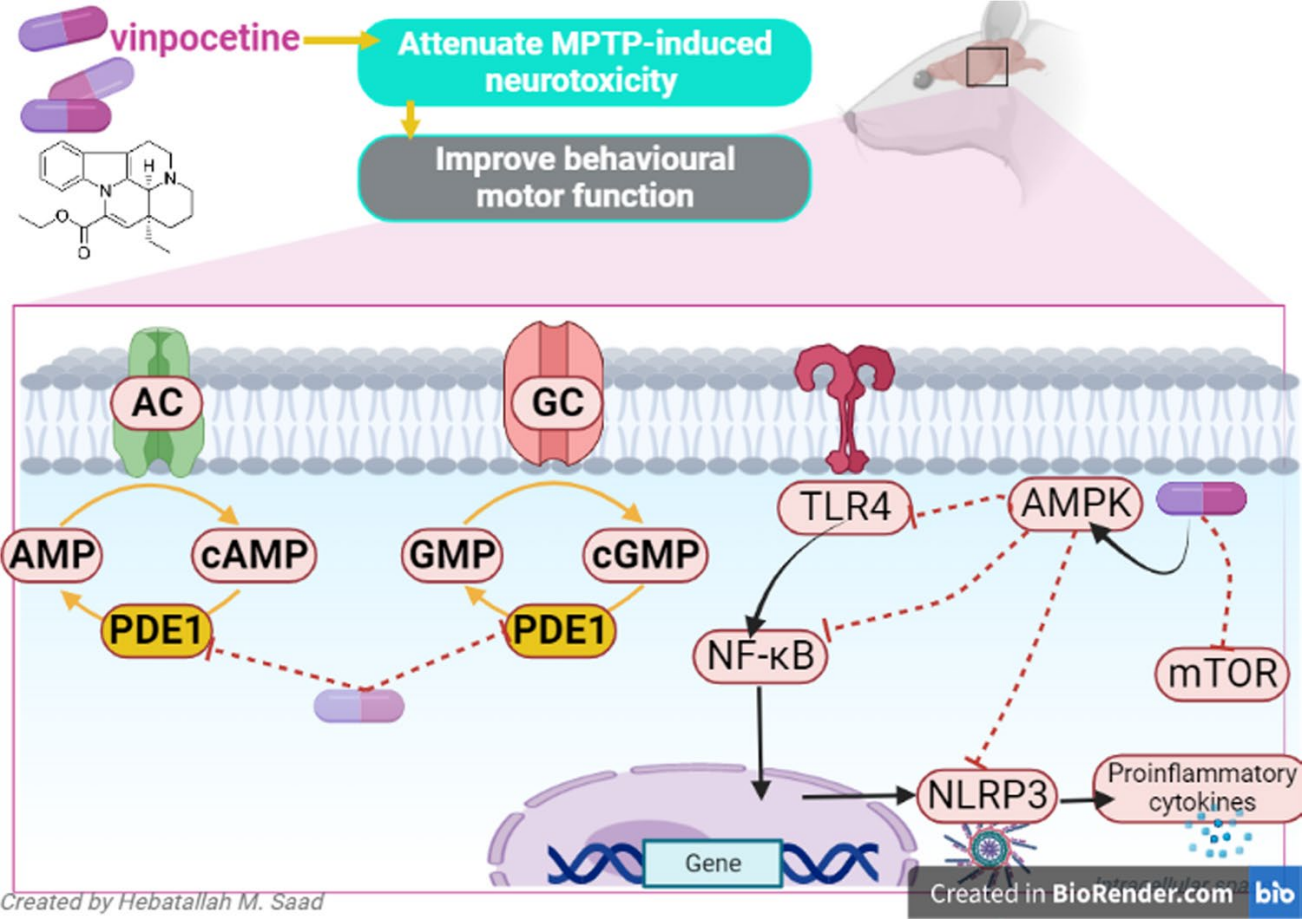
Role of vinpocetine (VPN) in Parkinson’s disease (PD): VPN improves PD through phosphodiesterase enzyme 1 (PDE-1) inhibition with increasing cyclic adenosine monophosphate (cAMP)/cyclic guanosine monophosphate (cGMP)signaling pathway. cAMP leads to antioxidant effects, while cGMP leads to anti-inflammatory effects with a reduction of 1-methyl-4-phenyl-1,2,3,6-tetrahydropyridine (MPTP)-induced neurotoxicity and development of motor severity in PD. Additionally, VPN attenuates the development of neuroinflammation via inhibtion of mTOR and the activation of adenosine monophosphate protein kinase (AMPK) that inhibit toll like receptor 4 (TLR4)/ nuclear factor kappa B (NF-κB)/ node like recptor pyrin 3 (NLRP3) inflammasome

PDEs are highly expressed in the striatum and SN and regulate dopamine signaling through cAMP/cGMP signaling (Nthenge-Ngumbau and Mohanakumar 2018). Several PDE families are highly expressed in the striatum including PDE1–4, PDE7, PDE9 and PDE10. There are growing evidence to suggest that these enzymes play a critical role in modulating cAMP-mediated dopamine signaling at the postsynaptic region. Therefore, it is clear that PDEs, given the broad range of subtypes and their varied tissue- and region-specific distributions, will be able to provide a range of possibilities as drug targets. There is no phosphodiesterase inhibitor currently approved for use against PD. The development of small molecule inhibitors against cyclic nucleotide PDE is a particularly hot area of investigation, and a lot of research and development is geared in this direction with major players in the pharmaceutical industry investing heavily in developing such potential drug entities (Nthenge-Ngumbau and Mohanakumar 2018). A recent experimental study confirmed that PDE7 is linked with the development of neurodegeneration and neuroinflammation (Morales-Garcia et al. 2020). Therefore, PDE inhibitors, through their neuroprotective effects, could be a new therapeutic strategy for treating PD. Garcia et al. (2014) observed that PDEA10 inhibitors might effectively manage PD and other brain neurodegenerative diseases. Interestingly, synthetic quinazoline derivatives produce a neuroprotective effect against the progression of PD through the inhibition of PDE1 (Laddha and Bhatnagar 2009). VPN improves PD neuropathology through PDE1 inhibition with a subsequent increase of the cAMP/cGMP signaling pathway. Therefore, increasing cAMP leads to antioxidant effects. In contrast, augmentation of cGMP by VPN leads to the development of motor severity in PD (Niccolini et al. 2015). Of note, striatal, and pallidal loss of PDE10A expression, which is associated with Parkinson’s duration and severity of motor symptoms and complications? PDE10A is an enzyme that could be targeted with novel pharmacotherapy, and this may help improve dopaminergic signaling and striatal output and therefore alleviates symptoms and complications of PD (Niccolini et al. 2015). Though, The severity of excessive daytime sleepiness in PD was associated with elevated PDE4 expression (Wilson et al. 2020). Therefore, VPN through inhibition of PDE1 improves cAMP/cGMP signaling in the dopaminergic neurons in the SN. However, none of the PDE inhibitors is approved for managing PD (Fig. 5).

### Glycogen synthase kinase 3β

Glycogen synthase kinase-3 (GSK-3) is a pleiotropic serine/threonine protein kinase found in almost all eukaryotes. It is structurally highly conserved and has been identified as a multifaceted enzyme affecting a wide range of biological functions, including gene expression and cellular processes. There are two closely related isoforms of GSK-3; GSK-3α and GSK-3β. The latter appears to play crucial roles in regulating the pathogenesis of diverse diseases, including neurodegenerative diseases including PD (Li et al. 2014). It has been shown that dysregulation of GSK-3β is associated with the pathogenesis of PD and by increasing accumulation of tau protein and production of α-synuclein (Li et al. 2014). GSK-3β has an antioxidant effect by inhibiting the generation of ROS. However, mutant GSK-3β triggers the degeneration of dopaminergic neurons in the SN causing PD (Li et al. 2014). Numerous apoptotic conditions can be facilitated by the GSK-3β signaling pathways. Studies have shown that GSK-3β inhibition protects the dopaminergic neurons from various stress-induced injuries, indicating the involvement of GSK-3β in PD pathogenesis (Li et al. 2014; Wang et al. 2007). However, the underlying mechanisms of the protective effect of GSK-3β inhibition on dopaminergic neurons in PD are not completely understood (Li et al. 2014; Wang et al. 2007). Multiple pathological events have been recognized to be responsible for the loss of dopaminergic neurons in PD, including mitochondrial dysfunction, oxidative stress, protein aggregation, and neuroinflammation (Wang et al. 2007). GSK-3β is a critical intermediate of MPTP neurotoxicity, and inhibition of GSK-3β may provide a novel strategy to treat PD (Wang et al. 2007). Thus, inhibition of dysregulated GSK-3β could be effective in reducing PD severity (Wang et al. 2007). Inhibition of GSK-3β attenuates the development of MPTP-induced PD in mice (Wang et al. 2007). VPN inhibits aluminum-induced cognitive impairment in rats by inhibiting the expression of GSK-3β (Ali et al. 2019). In addition, VPN mitigates ketamine-induced behavioral abnormalities by inhibiting GSK-3β (Ahmed et al. 2018). Therefore, VPN could effectively treat PD by inhibiting the expression of neuronal GSK-3β.

### Brain-derived neurotrophic factor

Brain-derived neurotrophic factor (BDNF) is a small dimeric protein, structurally related to nerve growth factor, which is abundantly and widely expressed in the adult mammalian brain (Murer et al. 2001). BDNF has been found to promote survival of all major neuronal types affected in PD, like hippocampal and neocortical neurons, cholinergic septal and basal forebrain neurons, and nigral dopaminergic neurons (Murer et al. 2001). BDNF is a potent inhibitor of apoptosis-mediated cell death and neurotoxin-induced degeneration of dopaminergic neurons. There is a growing body of evidence implicating BDNF in the pathogenesis of PD, suggesting it may eventually be used in the development of neuroprotective therapies for PD (Scalzo et al. 2010). A case-control study on 47 PD patients and 23 control subjects showed that serum BDNF serum level was significantly decreased in PD patients when compared with controls (Scalzo et al. 2010). Though, BDNF serum level was higher in the late stage of PD patients (Scalzo et al. 2010). Therefore, lower BDNF levels in the early stages of the disease may be associated with pathogenic mechanisms of PD, and an increase of BDNF levels with the progression of the disease may be a compensatory mechanism in more advanced stages of PD. A systematic review and meta-analysis revealed that PD patients had reduced serum BDNF levels compared to healthy controls, regardless of the presence of co-morbid depression. PD is at least equally effective in reducing serum BDNF levels as depression (Rahmani et al. 2019). Motor progression predicts serum BDNF downregulation in PD. Acute exercise improves motor function and depressive symptoms in PD probably via BDNF upregulation. The paradoxical rise in serum BDNF in advance of PD is probably compensatory in nature (Rahmani et al. 2019). Therefore, activating of BDNF signaling pathway in the early stage of PD may attenuate the progression of PD neuropathology. It has been reported that VPN can reverse the synaptic ultrastructure by regulating BDNF-related PSD-95 to alleviate schizophrenia-like deficits induced by ketamine in rats (Xu et al. 2019). The underlying mechanism is that VPN could increase cAMP and cGMP levels, thereby increasing the activity of cAMP and cGMP-dependent protein kinase A (PKA) and protein kinase G (PKG), which could promote the activation of many genes, such as BDNF (Swart et al. 2017). As well, VPN improves behavioral changes in autism spectrum disorder in rats by improvement of BDNF signaling (Luhach et al. 2022). Therefore, VPN through the improvement of BDNF signaling may attenuate PD neuropathology.

Taken together, VPN may improve PD by anti-inflammatory and antioxidant effects with modulation of various signaling pathways including NF-κB, NLRP3 inflammasomes, GSK-3β and BDNF.

## Conclusion

VPN has anti-inflammatory and antioxidant effects by inhibiting the expression of NF-κB and PDE-1. VPN is used in the management of dementia and stroke. VPN has protective and restorative effects against neuronal injury by reducing neuroinflammation and synaptic plasticity and improving cerebral blood flow. VPN protects dopaminergic neurons by reducing oxidative stress, lipid peroxidation, glutamate neurotoxicity, and regulation of Ca^+ 2^ overloads. Likewise, VPN can alleviate PD neuropathology through its anti-inflammatory, antioxidant, antiapoptotic and regulation of inflammatory signaling pathways. Moreover, VPN could effectively treat PD by inhibiting the expression of neuronal GSK-3β. VPN also attenuates neuroinflammation-induced PD by inhibiting the expression of inflammatory signaling NF-κB and the release of pro-inflammatory cytokines. VPN through inhibition of PDE1 improves cAMP/cGMP signaling in the dopaminergic neurons in the SN. This review indicated that VPN could be effective in the management of PD. However, preclinical and prospective studies are warranted to confirm the possible beneficial role of VPN in PD.

## Data Availability

Not applicable.
